# Modularly Programmable Nanoparticle Vaccine Based on Polyethyleneimine for Personalized Cancer Immunotherapy

**DOI:** 10.1002/advs.202002577

**Published:** 2021-01-06

**Authors:** Jutaek Nam, Sejin Son, Kyung Soo Park, James J. Moon

**Affiliations:** ^1^ Department of Pharmaceutical Sciences Biointerfaces Institute University of Michigan Ann Arbor MI 48109 USA; ^2^ Department of Biomedical Engineering Biointerfaces Institute University of Michigan Ann Arbor MI 48109 USA; ^3^ Department of Pharmaceutical Sciences Department of Biomedical Engineering Biointerfaces Institute University of Michigan Ann Arbor MI 48109 USA

**Keywords:** cancer vaccines, immunotherapy, nanoparticles, neoantigens

## Abstract

Nanoparticles (NPs) can serve as a promising vaccine delivery platform for improving pharmacological property and codelivery of antigens and adjuvants. However, NP‐based vaccines are generally associated with complex synthesis and postmodification procedures, which pose technical and manufacturing challenges for tailor‐made vaccine production. Here, modularly programmed, polyethyleneimine (PEI)‐based NP vaccines are reported for simple production of personalized cancer vaccines. Briefly, PEI is conjugated with neoantigens by facile coupling chemistry, followed by electrostatic assembly with CpG adjuvants, leading to the self‐assembly of nontoxic, sub‐50 nm PEI NPs. Importantly, PEI NPs promote activation and antigen cross‐presentation of antigen‐presenting cells and cross‐priming of neoantigen‐specific CD8^+^ T cells. Surprisingly, after only a single intratumoral injection, PEI NPs with optimal PEGylation elicit as high as ≈30% neoantigen‐specific CD8^+^ T cell response in the systemic circulation and sustain elevated CD8^+^ T cell response over 3 weeks. PEI‐based nanovaccines exert potent antitumor efficacy against pre‐established local tumors as well as highly aggressive metastatic tumors. PEI engineering for modular incorporation of neoantigens and adjuvants offers a promising strategy for rapid and facile production of personalized cancer vaccines.

## Graphical abstract

Modularly programmed, polyethyleneimine (PEI)‐based nanoparticle (NP) vaccines are developed for simple production of personalized cancer vaccines. PEI‐antigen conjugates and CpG adjuvants are mixed form nontoxic, sub‐50 nm PEI NPs. PEI NPs elicit strong, long‐lasting neoantigen‐specific CD8^+^ T cell response with potent antitumor efficacy against local as well as metastatic tumors. PEI NPs offer a promising strategy for personalized cancer vaccination.

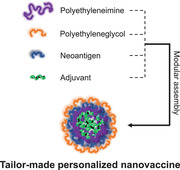

## 1 Introduction

Therapeutic cancer vaccination aims to activate and augment antitumor T cell immunity by providing antigenic and costimulatory signals to professional antigen‐presenting cells (APCs).^[^ ^1^ ^]^ In particular, neoantigens, produced by genetic alterations occurring in a tumor‐ and patient‐specific manner, can be highly immunogenic as neoantigens are entirely absent in normal cells, thus bypassing central T cell tolerance.^[^ ^2^ ^]^ Thus, amplifying neoantigen‐specific T cells using cancer vaccines offers a promising strategy for improving immunogenicity and selectivity of cancer vaccines.^[^ ^3^ ^]^ Indeed, neoantigen vaccines based on peptides have recently generated promising clinical outcomes in small cohorts of patients.^[^ ^4^ ^]^ Although these initial clinical trials provide strong rationale for further development of neoantigen cancer vaccines, these initial studies employing free soluble vaccines exhibited limited efficiency at generating neoantigen‐specific T cells, potentially due to rapid clearance of free antigens upon in vivo administration and poor codelivery of antigens and adjuvants to APCs.^[^ ^5^ ^]^

Nanoparticle (NP)‐based delivery systems have several advantages for cancer vaccination, including improved pharmacological properties, targeted delivery, and controlled and localized release of immunomodulatory agents for efficient modulation of specific immune cells.^[^ ^6^ ^]^ Various functional NPs based on liposomes, polymers, lipoprotein nanodiscs, and inorganic NPs have been employed to improve innate immune stimulation and induction of antitumor T cell responses,^[^ ^7^ ^]^ including personalized neoantigen cancer vaccines.^[^ ^8^ ^]^ However, many NP‐based vaccines generally involve complex synthesis steps and postmodification of NPs, thus presenting technical and manufacturing challenges. On the other hand, it is desirable to streamline the manufacturing process of neoantigen‐based vaccines so that simple, scalable, affordable production with short turnaround time is feasible for practical neoantigen‐based cancer vaccination in the clinic.^[^ ^9^ ^]^

**Figure 1:**
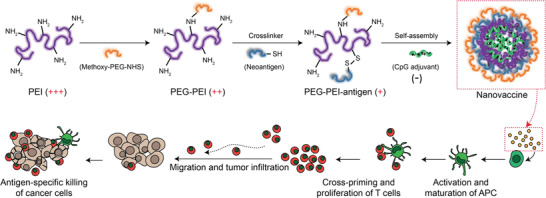
Schematic illustration of PEI‐based nanovaccine. PEI was sequentially modified with PEG and neoantigens via amide and disulfide bond, respectively. Then, polycationic PEI conjugates were self‐assembled with polyanionic CpG adjuvants through electrostatic interaction to form neoantigen nanovaccine. Diverse types of antigens and adjuvants can be incorporated into the complex allowing flexible and modular design for personalized cancer vaccines. The nanovaccine can increase the cellular uptake of neoantigens and adjuvants by APCs and promote activation and antigen cross‐presentation to effectively cross‐prime antigen‐specific T cells for robust antitumor immunity and antitumor efficacy.

Here, we designed a programmable neoantigen cancer vaccine that allows simple and facile modular assembly of defined antigens and adjuvants by exploiting the versatile functionality of polyethyleneimine (PEI) (**Figure** **1**). Furthermore, we sought to perform systemic investigation on PEI‐based vaccine system for promoting cellular uptake of neoantigens, activation of APCs, and cross‐priming of neoantigen‐specific T cell responses. Our vaccine consists of PEI‐antigen conjugates and CpG adjuvants that form compact nano‐condensates through electrostatic interaction between polycationic PEI and polyanionic CpG. PEI‐antigen is composed of neoantigen peptides conjugated to PEI via a disulfide bond that can be readily cleaved in the highly reductive intracellular environment, thereby promoting cross‐presentation by APCs.^[^ ^8b,d^ ^]^ Subsequently, PEI‐antigen conjugates are incubated with CpG to self‐assemble into nano‐sized particles for efficient codelivery of antigens and adjuvants to APCs – a prerequisite step for optimal T cell priming.^[^ ^10^ ^]^ Our approach to exploit the intrinsic charge property can avoid complex chemical and structural modifications and preserve immunological activities of antigens and adjuvants to achieve maximum potency.^[^ ^11^ ^]^ Importantly, we show polyethyleneglycol (PEG) modification as a simple yet powerful strategy to improve the PEI‐based nanovaccine for cellular uptake, activation, and antigen cross‐presentation of APCs, while eliminating inherent cytotoxicity associated with PEI. The optimized nanovaccines elicited robust priming of antigen‐specific CD8^+^ T cells and exerted strong antitumor efficacy against pre‐established local and metastatic tumors, demonstrating their potential for personalized cancer immunotherapy.

## 2 Results

### 2.1 PEGylation Reduces Cytotoxicity of PEI–Adpgk Conjugates and Produces Sub‐50 nm CpG Complex

**Figure 2:**
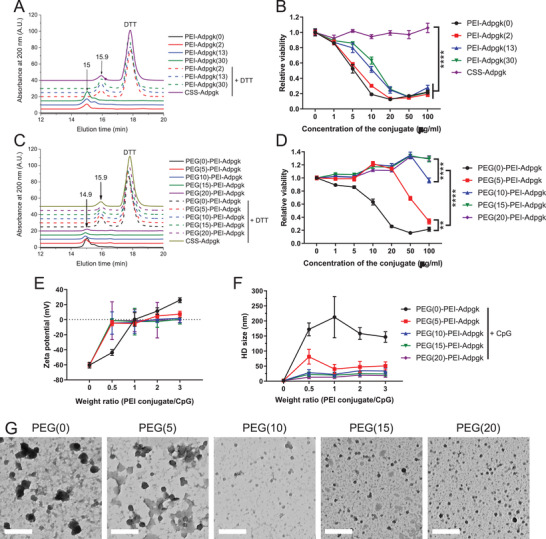
Synthesis and characterization of PEI conjugates and CpG‐containing nanovaccines. A–D) GPC spectra of A) PEI–Adpgk conjugates and C) PEG–PEI–Adpgk conjugates measured before and after 10 × 10^−3^ m DTT treatment, and B, D) their dose‐dependent cytotoxicity toward BMDCs assessed after 24 h incubation. The number denotes number of conjugated Adpgk per PEI for PEI–Adpgk conjugates and number of grafted PEG per PEI for PEG‐PEI–Adpgk conjugates. E) Zeta potential and F) hydrodynamic size of nanovaccines formed by adding CpG to PEG–PEI–Adpgk conjugates with varying weight ratio. G) TEM images of nanovaccines formulated at a weight ratio of 2 taken after 2% uranyl acetate staining for visualization of their morphology. Scale bars = 200 nm. The data show mean  ±  s.d. (*n*  =  5). **P  <  0.01 and ****P  <  0.0001, analyzed by two‐way ANOVA with Bonferroni multiple comparisons post‐test.

We prepared PEI‐antigen conjugates by employing an amine‐to‐sulfhydryl cross‐linker that bridges PEI and cysteine‐modified peptides through a reducible disulfide bond. As for the choice of antigen, we employed Adpgk peptide which is a neoantigen identified in murine MC38 colon carcinoma.^[^ ^3b^ ^]^ Specifically, the primary amine of PEI was grafted with the cross‐linker to create pyridyldithiol functional groups to which CSS‐Adpgk was conjugated to form PEI–Adpgk via disulfide linkage. The feeding amount of the cross‐linker and CSS‐Adpgk was varied to adjust the density of Adpgk peptide, and the PEI–Adpgk conjugates were analyzed by gel permeation chromatography (GPC) (**Figure** **2**A). PEI–Adpgk conjugates displayed strong absorption peaks for Adpgk peptide at ≈15 min, which was absent in plain PEI (labeled as PEI–Adpgk(0)). When the conjugates were treated with dithiothreitol (DTT) reducing agent, the elution time of PEI–Adpgk conjugates was delayed by ≈0.9 min, and their peaks coeluted with free CSS‐Adpgk + DTT. These results demonstrated successful conjugation of Adpgk peptide via reduction‐sensitive bond, which would allow for the release of intact peptides in a reducing environment. We prepared PEI–Adpgk conjugates with Adpgk/PEI molar ratios of 2, 13, and 30, as determined from the standard curve of CSS‐Adpgk + DTT and concentration of Adpgk released from DTT treatment of PEI–Adpgk (Figure S1, Supporting Information). We could not obtain higher Adpgk conjugation as it caused precipitation due to poor solubility in aqueous medium. Since APCs are the first line of immune cells that vaccine formulations should engage for priming antitumor T cell response, we examined PEI–Adpgk conjugates for potential cytotoxicity in bone marrow‐derived dendritic cells (BMDCs) (Figure 2B). As PEI is known to be cytotoxic,^[^ ^12^ ^]^ BMDCs incubated with plain PEI and PEI–Adpgk conjugates exhibited similar levels of cytotoxicity although we observed slightly reduced cytotoxicity for PEI–Adpgk conjugates with Adpgk/PEI ratio ≥ 13.

We sought to reduce the cytotoxicity of PEI–Adpgk by employing PEGylation. PEG–PEI–Adpgk conjugates were synthesized by unsaturated conjugation of methoxy poly(ethyleneglycol) propionic acid *N*‐hydroxysuccinimide (methoxy‐PEG‐NHS) to a portion of the primary amine of PEI, followed by the cross‐linker and CSS‐Adpgk conjugation as above. For the systemic investigation, we varied the degree of PEGylation by adjusting the stoichiometry of PEG:PEI to 5:1, 10:1, 15:1, or 20:1. The efficiency of PEG conjugation was nearly 100% for all cases as calculated from the unreacted free amine groups quantified using 2,4,6‐trinitrobenzene sulfonic acid (data not shown).^[^ ^13^ ^]^ PEG–PEI–Adpgk conjugates were also confirmed using GPC spectra, which showed similar ≈1 min peak shift after DTT treatment (Figure 2C), indicating stable conjugation of Adpgk peptides via disulfide linkage. The ratio of Adpgk:PEI was calculated to be 46, 43, 37, and 33 for PEG(5)‐, PEG(10)‐, PEG(15)‐, PEG(20)‐PEI–Adpgk conjugates, respectively. The more PEG grafted, the smaller number of Adpgk was conjugated to PEI, probably due to the steric hindrance of PEG. PEGylation dramatically improved biocompatibility of PEI–Adpgk conjugates with PEG/PEI ≥ 15 exhibiting no cytotoxicity up to 100 µg mL^−1^ and rather promoting cellular proliferation to some extent (Figure 2D). PEGylation was mainly responsible for the reduced cytotoxicity although Adpgk conjugation also partially contributed to it (Figure S2, Supporting Information).

We next investigated PEG–PEI–Adpgk conjugates formulated with CpG. The cationic PEI in PEG–PEI–Adpgk can allow electrostatic assembly and condensation of anionic CpG, confining antigens and adjuvants into nanoparticles (NPs). NPs were formulated by rapid mixing and 1 min incubation of CpG with PEG–PEI–Adpgk conjugates at weight ratios of PEG–PEI–Adpgk/CpG ranging 0.5–3. CpG had a zeta potential of −60 mV from its phosphorothioate backbone units; as the feed amount of PEG–PEI–Adpgk increased, the zeta potential of PEG–PEI–Adpgk/CpG NPs gradually increased toward more positive values (Figure 2E). As CpG was added to PEG(0)–PEI–Adpgk and PEG(5)–PEI–Adpgk, they underwent complete charge conversion to positive at weight ratio > 1, while the conjugates with PEG ≥ 10 remained nearly neutral. These results suggest charge compensation of CpG by PEG–PEI–Adpgk conjugates by electrostatic assembly and passivation of their surface by the nonionic PEG layer. Complete CpG condensation appeared to occur at the PEG–PEI–Adpgk/CpG weight ratio of 2, based on the zeta potential measurement. As shown by the dynamic light scattering (DLS) measurements, the hydrodynamic (HD) size of NPs generally did not change at PEG–PEI–Adpgk/CpG weight ratio ≥ 2, and at the weight ratio of 2, PEG(0)‐, PEG(5)‐, PEG(10)‐, PEG(15)‐, and PEG(20)‐PEI–Adpgk conjugates formed NPs with HD size of 158 ± 19, 47 ± 18, 35 ± 16, 25 ± 7, and 20 ± 6 nm, respectively (Figure 2F). The negative correlation between PEG density and HD size suggests that PEG passivation promotes formation of small NPs by enhancing their colloidal stability, which is in line with previous reports.^[^ ^14^ ^]^ We confirmed NP formation with transmission electron microscopy (Figure 2G), which showed the size profiles in alignment with the DLS measurements.

Overall, PEGylation significantly reduced cytotoxicity of PEI–Adpgk conjugates and stabilized their CpG nanocomplex, thereby generating sub‐50 nm NPs with a nearly neutral surface charge. The approach presented here allows the synthesis of well‐defined PEI‐antigen conjugates using facile conjugation chemistry. Subsequently, NPs can be readily produced in a few minutes by simple mixing and brief incubation with molecularly‐defined adjuvants. Thus, the PEI‐based NP system offers a promising manufacturing strategy for on‐demand production of personalized cancer vaccines with a quick turnaround. Based on the zeta potential and HD size measurements, we chose NPs formed at the PEG–PEI–Adpgk:CpG weight ratio of 2:1 for the subsequent studies.

### 2.2 PEGylation Enhances Cellular Uptake of CpG Nanocomplex and Promotes Activation and Antigen Presentation of BMDCs In Vitro

**Figure 3:**
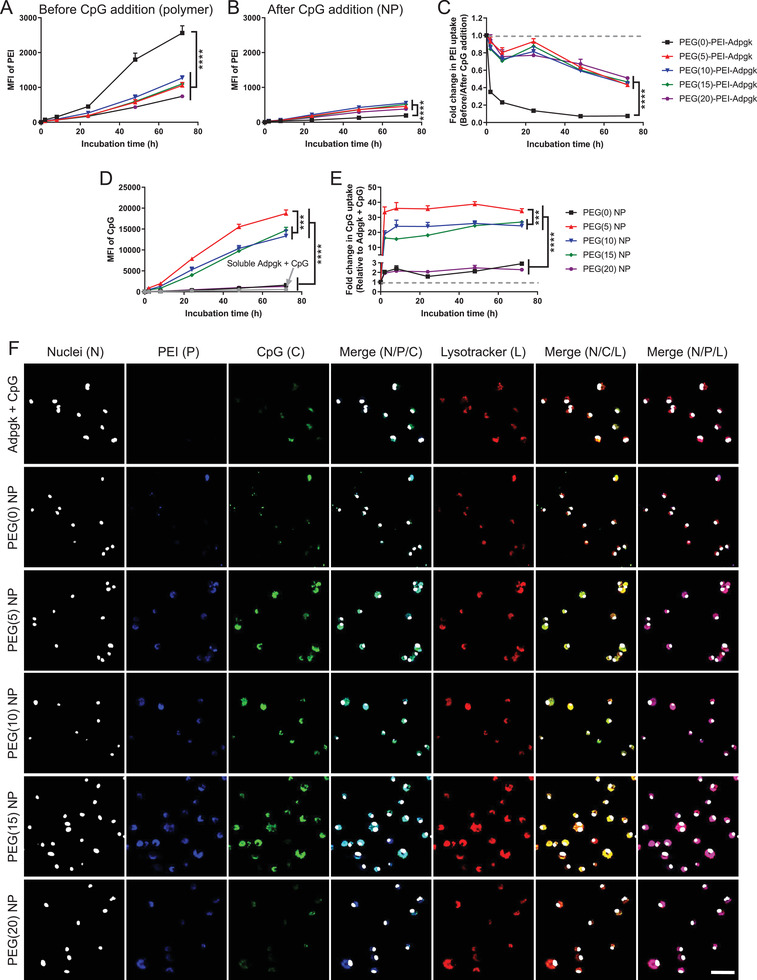
Uptake of nanovaccines by BMDCs. A–C) Time lapse uptake of PEG–PEI–Adpgk conjugates in the form of A) free polymer or B) their nanovaccines formulated by adding CpG measured over 3 days, and C) corresponding fold change in the uptake of PEG–PEI–Adpgk conjugates after CpG addition. D) Time lapse uptake of CpG and E) corresponding fold change in CpG uptake by nanovaccines, compared to soluble Adpgk + CpG. F) Confocal microscope images of BMDCs after 24 h incubation with soluble Adpgk + CpG or nanovaccine samples. Scale bar = 50 µm. The data show mean  ±  s.d. (*n*  =  6). ***P  <  0.001 and ****P  <  0.0001, analyzed by two‐way ANOVA with Bonferroni multiple comparisons post‐test.

Next, we sought to investigate how PEGylation impacts on the interactions between nanovaccines and BMDCs. PEG–PEI–Adpgk conjugates and CpG were separately tagged with distinct fluorophores, formulated into NPs, incubated with BMDCs, and visualized to track cellular uptake of each components over time. The doses of PEG–PEI–Adpgk and CpG were fixed at 2 and 1 µg mL^−1^, respectively. PEGylation decreased cellular uptake of PEI–Adpgk conjugates (without CpG), likely due to the antifouling and stealth feature of PEG (**Figure** **3**A).^[^ ^15^ ^]^ CpG‐mediated NP complexation decreased cellular uptake of PEG–PEI–Adpgk conjugates, compared with their respective free polymer form (Figure 3B), and in particular, PEG(0)–PEI–Adpgk exhibited the greatest extent of decrease than others (Figure 3C). Nevertheless, compared with soluble Adpgk + CpG, the nanovaccine formulation markedly enhanced cellular uptake of CpG (Figure 3D), with 30–40‐fold increase by PEG(5) NPs; 15–30‐fold increase by PEG(10) and PEG(15) NPs; and 2–3‐fold increase by PEG(0) and PEG(20) NPs (Figure 3E). Confocal microscopy images taken after 24 h incubation confirmed significant cellular uptake of both PEI–Adpgk conjugates and CpG for PEG(5), PEG(10), and PEG (15) NPs (Figure 3F). In addition, we observed colocalization of PEI–Adpgk conjugates and CpG in the endolysosomal compartments.

**Figure 4:**
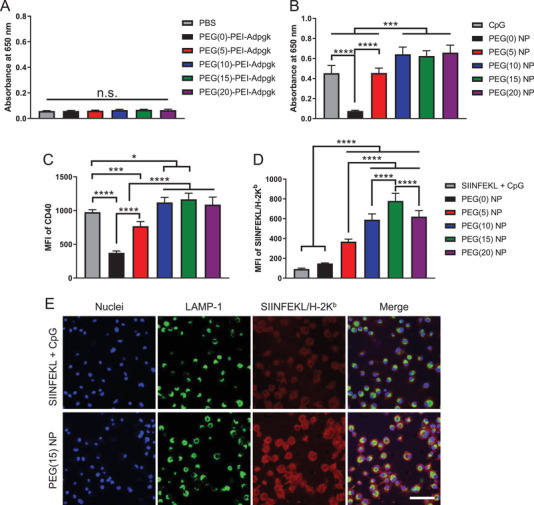
Induction of TLR9‐mediated immune stimulation and antigen cross‐presentation by nanovaccines. A,B) HEK‐Blue TLR9 cells were incubated with A) free polymer form of PEG–PEI–Adpgk conjugates or B) their nanovaccines with CpG, and induction of TLR9 signaling cascade was quantified using 650 nm absorbance. Upregulation of C) CD40 and D) SIINFEKL/H‐2K^b^ expression by BMDCs after 24 h incubation with SIINFEKL + CpG or SIINFEKL nanovaccines. E) Confocal microscope images of BMDCs incubated with SIINFEKL + CpG or PEG(15) NP of SIINFEKL nanovaccine. Scale bar = 50 µm. The data show mean  ±  s.d. (*n*  =  6). *P  <  0.05, ***P  <  0.001, and ****P  <  0.0001, analyzed by one‐way ANOVA with Bonferroni multiple comparisons post‐test.

Having shown the robust uptake of nanovaccine, we next investigated activation and antigen presentation of BMDCs. We examined nanovaccine‐mediated activation of Toll‐like receptor (TLR)‐9 using a HEK‐Blue TLR‐9 reporter cell line. When incubated with HEK‐Blue TLR‐9 cells, PEG–PEI–Adpgk conjugates induced only baseline signal, whereas CpG promoted strong activation of HEK‐Blue TLR‐9 cells, indicating TLR‐9 activation by CpG (**Figure** **4**A, B). Whereas PEG(0) NPs showed only a baseline response, PEGylation of NPs significantly elevated TLR9 activation, with PEG ≥ 10 inducing stronger response than free CpG.

Next, we examined how NP formulation impacts antigen presentation by BMDCs. To study this, we employed a model antigen, SIINFEKL peptide, which is an immunodominant MHC‐I epitope from ovalbumin. PEG‐PEI‐SIINFEKL conjugates were synthesized and confirmed using GPC analysis as in Figure 2 (Figure S3, Supporting Information). SIINFEKL nanovaccines formulated with CpG at a weight ratio of 2 were incubated with BMDCs for 24 h, and BMDCs were analyzed for maturation and antigen presentation. Upregulation of CD40, CD80, CD86 costimulatory marker on BMDCs (Figure 4C; Figure S4, Supporting Information) followed a similar pattern as PEG density‐dependent increase in TLR‐9 activation (Figure 4B), suggesting CpG‐mediated BMDC activation. In addition, PEG density also affected antigen presentation on BMDCs, as measured by monoclonal antibody against SIINFEKL/H‐2k^b^ (pMHC) complex (Figure 4D). NPs with higher PEG density generally increased antigen presentation, with PEG(15) NPs inducing 8.4‐fold higher pMHC level than soluble SIINFEKL + CpG (Figure 4D). Confocal microscopy also confirmed robust pMHC display on BMDCs treated with PEG(15) NPs, compared with soluble SIINFEKL + CpG control (Figure 4E). In addition, pMHC was mainly localized on the cell surface without much overlap with late endosomes/lysosomes stained with lysosomal associated membrane protein 1 (LAMP‐1) (Figure 4E). As PEI‐antigen conjugates and CpG were mainly localized in endo‐lysosomes (Figure 3F), these results suggest that the nanovaccine promotes intracellular delivery of antigens and CpG and the subsequent steps of cross‐presentation, including the intracellular processing of peptide antigen, MHC‐loading of epitopes, and trafficking of pMHC to the cell surface.^[^ ^16^ ^]^ Without CpG, PEG‐PEI‐SIINFEKL conjugates in the form of free polymers exhibited decreased CD80, CD86, CD40, and pMHC expression as the PEG density was increased (Figure S5, Supporting Information), possibly due to the decreased cellular uptake. This is an opposite trend from the case of nanovaccines, which suggests a unique beneficial role of PEGylation for nanovaccine. Overall, PEGylation on PEI‐antigen/CpG nanovaccines plays a vital role in cellular uptake, adjuvant activity, and antigen cross‐presentation, and high PEG density are generally favored for activation of DCs.

### 2.3 PEGylation Reduces Tumor Retention of Nanovaccine but Elicits Strong Immune Activation in Local Tumor‐Draining Lymph Nodes In Vivo

**Figure 5:**
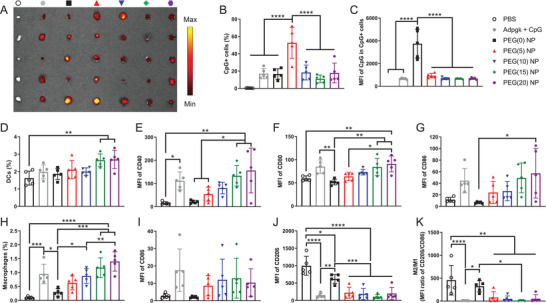
Tumor retention of the nanovaccine and immune activation in tumor‐draining LNs. A) Tumor retention of vaccines composed of various forms of Adpgk peptides and AF647‐CpG was visualized using ex vivo IVIS imaging after 24 h of intratumoral injection. Quantitative analysis of B) CpG+ cells and C) corresponding MFI of CpG in CpG+ cells in tumors. D–K) Tumor‐draining inguinal LNs were analyzed for the number and activation of D‐G) DCs and H–K) macrophages. The data show mean  ±  s.d. (*n*  =  5). *P  <  0.05, **P  <  0.01, ***P  <  0.001, and ****P  <  0.0001, analyzed by one‐way ANOVA with Bonferroni multiple comparisons post‐test.

Next, we investigated PEGylation‐dependent cellular uptake of nanovaccines in vivo. Tumor tissue consists of a variety of cells tightly organized in a confined volume, and thus provides a suitable biological model for studying complex cellular interactions. Adpgk nanovaccine was tested in a murine tumor model of MC38 colon carcinoma.^[^ ^3b^ ^]^ We established MC38 colon carcinoma subcutaneously on the right flank of C57BL/6 mice, and vaccines composed of Adpgk peptides and Alexa Fluor 647 (AF647)‐tagged CpG were administered directly into tumors. The fluorescence intensity of AF647‐CpG measured ex vivo after 24 h revealed that PEG(0) and PEG(5) NPs enhanced tissue retention of CpG (**Figure** **5**A), probably due to positive surface charges (Figure 2E). Flow cytometry‐based analysis of tumor tissues indicated that PEG(5) NPs were broadly distributed in a larger population of cells, whereas cellular uptake of PEG(0) NPs was mainly restricted to a small subset of cells that internalized NPs to a greater extent (Figure 5B,C). PEG(0) NPs appeared to be rapidly captured by cells at the injection site with limited distribution in the tumor tissues, whereas PEG(5) NPs exhibited increased distribution within the tumor tissues, probably due to the PEG passivation layer. CpG was mainly internalized by tumor cells and macrophages regardless of the formulations (Figure S6, Supporting Information).

Tumor‐draining lymph nodes (TDLNs) are critical sites where T‐cells are primed for immune activation against tumors.^[^ ^17^ ^]^ Therefore, we analyzed DCs and macrophages in inguinal TDLNs after intratumoral administration of NPs. First, we confirmed that AF647 conjugation did not compromise the adjuvanticity of CpG using BMDCs in vitro (Figure S7, Supporting Information). PEG(15) and PEG(20) NPs enriched DCs in TDLNs and elevated their expression of CD40, CD80, and CD86 costimulatory markers (Figure 5D–G). In contrast, PEG(0), PEG(5), and PEG(10) NPs induced weaker activation of DCs in TDLNs (Figure 5D–G), suggesting that PEG density on NPs plays a crucial role in DC activation in TDLNs. Similar PEG‐dependency was observed for the number of macrophages in TDLNs (Figure 5H), with PEG(15) and PEG(20) NPs significantly increasing macrophages compared with PEG(0) NPs. Compared with PBS and PEG(0) NP, PEGylated NPs as well as the soluble vaccine group upregulated CD86 and downregulated CD206 on macrophages in TDLNs (Figure 5I,J), resulting in a decreased ratio of M2/M1‐like macrophages (Figure 5K).^[^ ^18^ ^]^ We observed similar activation of DCs and macrophages in tumor‐draining axillary LNs, but not in contralateral non‐tumor‐draining inguinal or axillary LNs (Figures S8 and S9, Supporting Information). These results show that a high degree of PEGylation potentiates the performance of nanovaccines upon cellular entry despite the reduction in direct cellular association, which is in agreement with in vitro results.

### 2.4 Antitumor Immune Response of Nanovaccine against Pre‐Established Local Tumor

**Figure 6:**
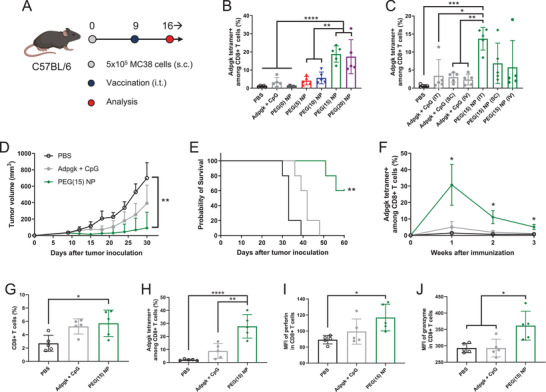
Antitumor immune response of nanovaccine against pre‐established local tumors. A) Schematic of treatment regimen. B,C) Adpgk‐specific CD8^+^ T cells in blood were analyzed after B) intratumoral injection of various vaccine formulations or C) administration of Adpgk + CpG versus PEG(15) NP via different routes of vaccination. MC38 tumor‐bearing mice were treated by intratumoral administration of Adpgk + CpG versus PEG(15) NP on day 9, and D) tumor growth and E) animal survival were monitored. F) Adpgk‐specific CD8^+^ T cells in blood observed over 3 weeks after single immunization. Tumor microenvironment analysis for the frequency of G) CD8^+^ T cells and H) Adpgk‐specific CD8^+^ T cells, mean fluorescence intensity (MFI) of I) perforin and J) granzyme in total CD8^+^ T cells. The data show mean  ±  s.d. (*n*  =  5). *P  <  0.05, **P  <  0.01, ***P  <  0.001, and ****P  <  0.0001, analyzed by one‐way (B,C,G,H,I,J) or two‐way (D,F) ANOVA with Bonferroni multiple comparisons post‐test, or by E) log‐rank (Mantel–Cox) test.

Having shown the robust activation of DCs and macrophages in TDLNs, we next examined the potency of nanovaccines for priming antitumor T cell response. C57BL/6 mice were subcutaneously inoculated with MC38 cells, administered with Adpgk nanovaccines or soluble Adpgk + CpG on day 9 via intratumoral injection, and analyzed for antitumor immune responses (**Figure** **6**A). PEGylated nanovaccines induced robust priming of antigen‐specific CD8^+^ T cells in the systemic circulation, as measured by Adpgk tetramer staining of peripheral blood mononuclear cells (PBMCs) after 7 days of vaccination (Figure 6B). Surprisingly, with only a single injection, PEG(15) and PEG(20) NPs elicited potent neoantigen‐specific CD8^+^ T cell responses against Adpgk, with 5–6‐fold higher tetramer+ CD8^+^ T cells than soluble Adpgk + CpG (19 ± 4.5 and 17 ± 9.4% vs 3.3 ± 2.5%, *P* < 0.0001, Figure 6B). In contrast, PEG(5) and PEG(10) NPs induced comparable CD8^+^ T cell responses with soluble Adpgk + CpG, while PEG(0) NP had barely detectable response (Figure 6B).

Based on strong CD8^+^ T cell response induced by PEG(15) NPs, we focused on PEG(15) NPs and examined how the route of immunization impacts T cell responses. After 9 days of MC38 tumor inoculation, tumor‐bearing mice were administered with PEG(15) NPs via intratumoral, subcutaneous (s.c.), or intravenous (i.v.) routes, which resulted in elicitation of 14 ± 3.1, 6.9 ± 5.6, and 5.8 ± 7.4% Adpgk‐specific CD8^+^ T cell response, respectively, on day 16 (Figure 6C). In contrast, soluble Adpgk + CpG induced only 2–4% CD8^+^ T cell responses regardless of the injection routes. Intratumoral vaccination can be a promising cancer immunotherapy as it can elicit strong antitumor immunity without overt systemic exposure of the vaccines. In fact, there are currently a number of clinical trials evaluating direct intratumoral injection of immunotherapies.^[^ ^19^ ^]^ Based on these results and considerations, we chose intratumoral administration with PEG(15) NPs for the subsequent antitumor efficacy studies.

C57BL/6 mice were inoculated with MC38 tumor cells on day 0, and a single intratumoral injection of PEG(15) NP was given on day 9. PEG(15) NP effectively suppressed tumor growth (Figure 6D) and eliminated established tumors in 60% mice, leading to significant survival benefit compared with other groups (*P* < 0.01, Figure 6E). In contrast, soluble Adpgk + CpG had only a modest effect with all treated mice succumbing to tumors before day 50. Importantly, a single intratumoral administration of PEG(15) NP led to potent, systemic antitumor CD8^+^ T cell response, achieving up to ≈30% Adpgk‐tetramer+ CD8^+^ T cell response and sustaining elevated CD8^+^ T cell response over 3 weeks (*P* < 0.05, Figure 6F), whereas the soluble vaccine group induced weak and transient CD8^+^ T cell response.

Systemically activated CD8^+^ T cells need to migrate and infiltrate into the tumor bed in order to recognize and eradicate cancer cells.^[^ ^20^ ^]^ To investigate tumor homing and cytotoxic activity of CD8^+^ T cells, we analyzed the tumor microenvironment after 7 days of PEG(15) NP treatment. PEG(15) NPs promoted tumor infiltration of CD8^+^ T cells (Figure 6G), with significantly increased frequency of Adpgk‐specific CD8^+^ T cells (28 ± 9.0%), representing 14‐ and 3.2‐fold increases over PBS and soluble Adpgk + CpG, respectively (Figure 6H). Although soluble Adpgk + CpG slightly elevated the frequency of CD8^+^ T cells in the tumor microenvironment, only a small subset of intratumoral CD8^+^ T cells was specific to Adpgk peptide, with no statistical difference from that of PBS‐treated mice (Figure 6G,H). Intratumoral CD8^+^ T cells primed with PEG(15) NPs had high expression levels of perforin and granzyme (Figure 6I,J), indicating their cytotoxic potential. On the other hand, we observed minimal activation of CD4^+^ T cells and NK cells (Figure S10, Supporting Information). Taken together, these results demonstrate that the nanovaccines can induce a robust and durable antitumor response by promoting clonal expansion and tumor infiltration of antitumor CD8^+^ T cells.

### 2.5 Nanovaccine against Highly Aggressive and Metastatic Tumor Model

**Figure 7:**
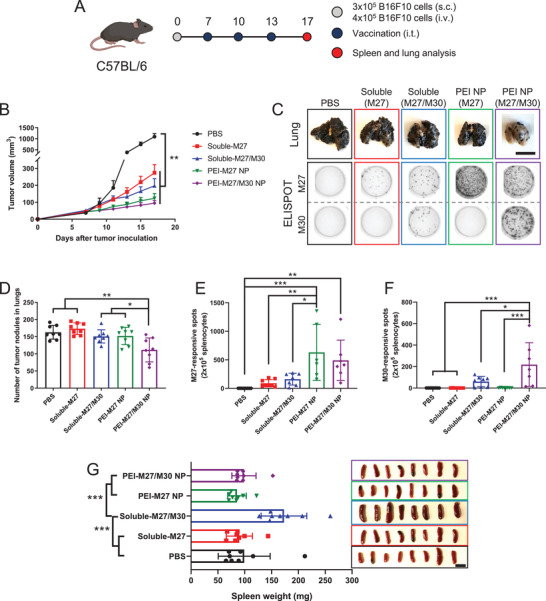
Antitumor immune response of nanovaccine against highly aggressive, disseminated B16F10 melanoma. A) Schematic of treatment regimen. B) Tumor growth curves of subcutaneous flank B16F10 tumors. C) Representative images of lungs and ELISPOT wells. ELISPOT assay was performed after restimulation of splenocytes with M27 or M30. Quantitative analysis of D) lung tumor nodules and ELISPOT counts against E) M27 or F) M30 performed on day 17. G) Weight and images of spleens for assessement of splenomegaly. Scale bars = 1 cm. The data show mean  ±  s.d. (*n*  =  8). *P  <  0.05, **P <  0.01, and ***P  <  0.001, analyzed by D–G) one‐way or B) two‐way ANOVA with Bonferroni multiple comparisons post‐test.

Finally, we sought to evaluate the therapeutic potential of the nanovaccines using B16F10 melanoma, which is a highly aggressive model with poor immunogenicity. To mimic late stage, advanced cancer, we established B16F10 melanoma in both s.c. flank and lung tissues; C57BL/6 mice were inoculated with 3 × 10^5^ B16F10 cells at s.c. flank as well as 4 × 10^5^ B16F10 cells via i.v. administration, leading to the establishment of s.c. flank tumor and lung metastatic nodules (**Figure** **7**A). Antitumor efficacy of nanovaccines was examined against both local tumors and disseminated metastases after the vaccine formulations were administered directly into the s.c. flank tumors only. As this model is highly aggressive, we vaccinated animals three times on days 7, 10, and 13. In addition, we utilized recently reported neoantigens identified in B16F10 cells, namely MHC I‐restricted M27 and MHC II‐restricted M30 neoepitopes, in order to study the effect of combining MHC‐I epitope with MHC‐II epitope.^[^ ^3a^ ^]^ PEG(15)‐PEI‐M27 and PEG(15)‐PEI‐M30 were synthesized following the established protocol and confirmed using high‐performance liquid chromatography (Figure S11, Supporting Information). CpG was added to PEG(15)‐PEI‐M27 or the mixture of PEG(15)‐PEI‐M27 and PEG(15)‐PEI‐M30 conjugates, leading to the formation of PEI‐M27 NP and PEI‐M27/M30 NP, respectively. Both PEI NPs exhibited similar HD size and zeta potential with nearly neutral surface charge; HD size was measured as 29 ± 8.6 nm and 27 ± 8.2 nm, and zeta potential 6.0 ± 4.7 mV and −1.7 ± 3.9 mV for PEI‐M27 NP and PEI‐M27/M30 NP, respectively.

Both PEI‐M27 NP and PEI‐M27/M30 NP treatment groups potently inhibited the growth of primary s.c. flank tumors compared with PBS (*P* < 0.01, Figure 7B) although their antitumor effects were not statistically significant from the soluble vaccine group, probably due to the aggressive nature of this B16F10 model. Importantly, PEI‐M27/M30 NP treatment exerted potent systemic antitumor efficacy against B16F10 metastasis, leading to a significantly decreased number of lung metastatic nodules by day 17 (Figure 7C,D). In contrast, all other treatment groups had similar number of lung metastatic nodules as the PBS control. Analysis of splenocytes using interferon (IFN)‐*γ* enzyme‐linked immunospot (ELISPOT) assay showed that PEI‐M27 NP and PEI‐M27/M30 NP significantly enhanced antigen‐specific T cell responses against MHC‐I‐restricted M27 and MHC‐II‐restricted M30 neoepitopes (Figure 7C,E,F). Soluble formulations induced markedly lower antigen‐specific T cell responses. These results suggested that CD8^+^ T cell response against M27 neoepitope was largely sufficient for suppressing local B16F10 tumors, whereas systemic inhibition of metastasis required both antitumor CD8^+^ and CD4^+^ T cells. Interestingly, soluble‐M27/M30 treatment induced splenomegaly indicative of systemic inflammation,^[^ ^5a^ ^]^ whereas PEI‐M27/M30 NP and all other treatments showed no change, compared to the PBS control (Figure 6G).

Overall, these results demonstrate that nanovaccines tailored for eliciting a broad spectrum of T cell responses against multiple neoepitopes could effectively treat highly aggressive local and metastatic tumors, while mitigating acute systemic side effects associated with soluble vaccine treatment.

## 3 Discussion

PEI has been widely exploited as a gene transfection agent as it can form positively charged nanoscale complex with DNA or RNA oligonucleotides to promote their cellular uptake and expression.^[^ ^21^ ^]^ In addition, PEI can stimulate immune activation by triggering release of “danger signals” or “damage‐associated molecular patterns” as the result of cellular stress and damage caused by its cytotoxic actions.^[^ ^12^, ^22^ ^]^ The ability of PEI to induce inherent immune stimulation and efficient cellular transfection encouraged its development for vaccine applications associated with the delivery of protein‐ or DNA‐based antigens. However, previous studies mostly utilized PEI‐based vaccines for treating infectious disease with antibody response,^[^ ^23^ ^]^ while a handful of cancer applications indicated sub‐optimal intrinsic adjuvanticity of PEI for eliciting antitumor T cell response.^[^ ^24^ ^]^ This has been attributed in part to type 2 T helper cell (Th2)‐biased immune activation by PEI, which triggers inflammasome activation and humoral immunity rather than cellular immunity—a crucial criterion for successful cancer vaccination.^[^ ^23a,b^ ^]^ In addition, transfection of host bystander cells and subsequent cytotoxicity by PEI have been reported to activate T cells against self‐antigens, potentially causing immune‐related adverse events.^[^ ^22^, ^25^ ^]^ In this work, we sought to take advantage of the versatile functionality of PEI for delivery of antigens and adjuvants, while eliminating inherent cytotoxicity of PEI that has hampered cancer vaccine applications. Here, we have shown that PEGylation of PEI formulations significantly decreased cytotoxicity of PEI, while also improving the performance of PEI to deliver exogenous Th1‐favored CpG adjuvant along with neoantigens in a spatiotemporally concerted manner. The optimized PEI‐based nanovaccines generated robust antigen‐specific T cells with a magnitude significantly greater than previously reported PEI‐based vaccines,^[^ ^23^, ^24^ ^]^ suggesting new engineering opportunities of PEI‐based vaccines for personalized cancer immunotherapy.

**Figure 8:**
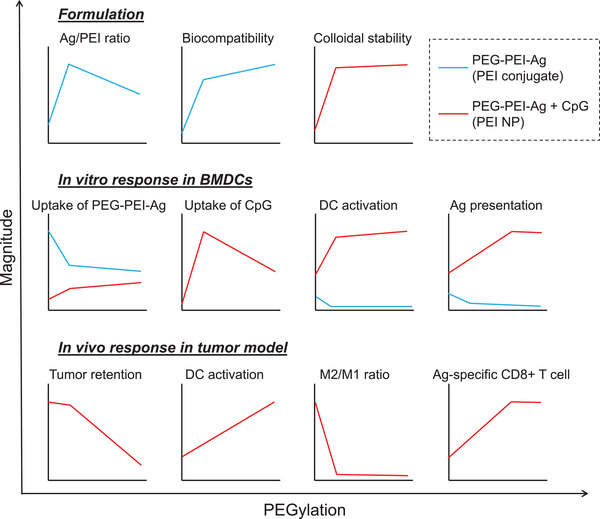
Summary of the impact of PEGylation on PEG‐PEI‐Ag formulation, in vitro DC activation, and in vivo immune activation.

PEG conjugation completely abolished cytotoxicity of PEI at stoichiometry of PEG/PEI ≥ 15, which is in line with previous reports.^[^ ^14^, ^26^ ^]^ In addition, PEG can serve as a uncharged spacer unit that provides steric stabilization, decreases nonspecific cellular uptake, and improves in vivo performance for PEI and its nano‐complex.^[^ ^27^ ^]^ PEGylation allowed for the formation of sub‐50 nm small NPs that significantly enhanced uptake of antigen and adjuvant by APCs. In particular, the uptake of CpG was greatly improved by the nanovaccine formulation, which could be attributed to gaining positive charges from the PEI‐antigen conjugate; in return, this caused decreased cellular uptake of PEI‐antigen conjugate with the loss of positive charges. Nonetheless, compared with non‐PEGylated PEI‐antigen/CpG nanocomplex, PEGylated PEI‐antigen/CpG nanovaccines increased uptake of both antigen and adjuvant, presumably due to PEG‐mediated surface passivation and enhanced colloidal stability. More importantly, the degree of PEGylation had a significant impact on immunological activity of nanovaccines, with higher PEG generally potentiating the vaccine efficacy regardless of the extent of cellular uptake. In vitro, this was clearly demonstrated with PEG(20) NP. Compared with NP formed with lower PEG densities, PEG(20) NP induced the least cellular uptake of PEI‐antigen and adjuvant (Figure 3); nevertheless, PEG(20) NP promoted robust TLR‐9 signaling (Figure 4B), upregulation of costimulatory markers (Figure 4C), and antigen cross‐presentation by DCs (Figure 4D). A similar observation was made in our in vivo studies. Activation of DCs and macrophages in TDLNs (Figure 5D–K) was increased with higher degree of PEGylation although PEGylation reduced cellular association of NPs in the tumor tissues (Figure 5A–C). PEG can not only passivate the surface of nanovaccines but also insert a charge‐inert layer during the assembly of PEI‐antigen and CpG that weakens electrostatic interaction. We speculate that PEGylation serves multi‐purposes; surface‐displayed PEG reduces nonspecific cellular uptake while inner PEG layer is thought to facilitate dissociation of the nanocomplexes in the sub‐cellular compartments. Efficient liberation of compactly packed nanocomplexes within target cellular compartments is a prerequisite for immune activation, serving as a crucial factor that governs the efficacy of nanovaccines and antitumor T cell responses.^[^ ^28^ ^]^ Nonetheless, there exists an optimal level of PEGylation for balancing cellular uptake and unpacking of nanocomplexes, as demonstrated by comparable in vitro and in vivo immune activation and T cell responses induced by PEG(15) NP and PEG(20) NP (Figures 4, 5, 6) despite significant lower cellular uptake of PEG(20) NP (Figure 3). In contrast, cellular uptake was directly associated with the activity of non‐CpG‐complexed free PEI‐antigen polymers, with PEGylation decreasing cellular uptake and subsequent activation and antigen presentation of BMDCs in vitro (Figure 3A; Figure S5, Supporting Information). Overall, these results suggest that immunological activity of nanovaccine is mainly limited by steric restriction of antigens and adjuvants, which could be improved by PEGylation that facilitates unpacking and liberation of antigens and adjuvants from the nanocomplex. The impact of PEGylation on the various aspects of formulation and performance of PEI‐based vaccine is summarized in **Figure** **8**.

The optimized nanovaccines allowed a greater amount of antigens and adjuvants to gain entry into cells than soluble vaccines, suggesting that the NP formulation promotes endocytosis/phagocytosis by DCs.^[^ ^29^ ^]^ We found that nanovaccines were located in endo‐lysosomal compartments (Figure 3F), leading to efficient triggering of the TLR‐9 signaling pathway and licensing of DCs for antitumor T cell responses (Figure 4).^[^ ^30^ ^]^ PEI has been known to mediate endosomal escape and cytosolic drug delivery by trapping endosomal protons, termed proton sponge effect.^[^ ^31^ ^]^ We speculate that the amount of PEI used for in vitro study (2 µg mL^−1^) was not sufficient to induce endosomal rupture via proton sponge effect. Nonetheless, we observed efficient cross‐presentation of endo‐lysosomally delivered antigens (Figure 4D,E). Thus, PEI NP‐mediated synchronous delivery of antigen and CpG to the endo‐lysosomal compartment could efficiently license DCs for cross‐priming of T cells. We speculate that PEI NP‐mediated antigen processing and MHC class I presentation occurs in endocytic compartments via vacuolar pathways,^[^ ^32^ ^]^ which could be further augmented by endosomal TLR signaling^[^ ^10a^ ^]^ and phagosomal MHC I delivery.^[^ ^33^ ^]^ Indeed, clonal expansion of antigen‐specific CD8^+^ T cells elicited by nanovaccines of varying PEG density (Figure 6B) followed the pattern of activation and maturation of DCs examined in vitro (Figure 4C) and in vivo (Figure 5E–G), supporting the link between DCs and T cells. Soluble vaccine induced significantly lower antigen‐specific CD8^+^ T cells than the nanovaccine (Figures 6 and 7) despite substantial induction of costimulatory markers on DCs (Figure 5). This suggests that soluble vaccines, which suffer from limited codelivery of antigens and adjuvants to the endo‐lysosomes (Figure 3D–F), have a poor antigen cross‐presentation as a major limitation for cancer vaccines (Figure 4D).^[^ ^10^ ^]^ We speculate that antigen availability may also be linked to the superiority of intratumoral vaccination to other administration routes (Figure 6C). Serving as an in situ antigen source, tumor tissue could supply endogenous tumor antigens that could be captured by or drained together with the vaccines after intratumoral injection, increasing antigen availability for vaccine‐primed DCs.^[^ ^34^ ^]^ Intratumoral injection of nanovaccines can also offer safe cancer immunotherapy by mitigating the systemic inflammation associated with the soluble vaccine (Figure 7G). With the optimal formulation and administration route, the nanovaccine developed in this study elicited remarkable CD8^+^ T cell responses and exerted robust antitumor efficacy in multiple murine tumor models, including advanced metastatic melanoma.

## 4 Conclusion

We have developed a personalized cancer vaccine based on PEI that allows nanoscale assembly of neoantigens and adjuvants with facile chemical modification and simple electrostatic interaction. The nanovaccine promoted activation and antigen cross‐presentation of APCs with efficient codelivery of immunologically active neoantigens and adjuvants, eliciting robust antitumor T cell immunity and antitumor efficacy against pre‐established local and metastatic tumors. Our approach allows modular incorporation of neoantigens and ajuvants for rapid and facile production of potent cancer nanovaccines. Our approach outlined here may offer a promising strategy for personalized cancer vaccination.

## 5 Experimental Section

##### Reagents and Instruments

Polyethyleneimine (PEI, branched, *M*_w_ 25000), 3‐(2‐pyridyldithio)propionic acid *N*‐hydroxysuccinimide ester (SPDP) were obtained from Sigma‐Aldrich. Methoxy poly(ethyleneglycol) propionic acid *N*‐hydroxysuccinimide (Methoxy‐PEG‐NHS, *M*_w_ 5000) was purchased from Nanocs. CpG1826 was obtained from Integrated DNA Technology. Antigen peptides used in this study were synthesized by Genemed Synthesis, which include epitopes of ovalbumin peptide SIINFEKL and CSSSIINFEKL, neo‐epitopes of MC38 colon carcinoma ASMTNMELM (Adpgk) and CSSASMTNMELM (CSS‐Adpgk), neo‐epitopes of B16F10 melanoma LCPGNKYEM (M27), VDWENVSPELNSTDQ (M30), and CSSVDWENVSPELNSTDQ (CSS‐M30). All other reagents were received from Fisher scientific unless otherwise indicated. UV–Vis absorption and fluorescence spectra were obtained using BioTek synergy neo microplate reader. GPC and HPLC were performed using Shimadzu HPLC system equipped with TSKgel G3000SWxl column (Tosoh Bioscience LLC) and Jupiter® C18 LC Column (Phenomenex), respectively. TEM images were acquired using JEOL 1400‐plus. Hydrodynamic size and zeta potential were measured using Zetasizer Nano ZSP (Malvern Panalytical). Flow cytometry was performed using ZE5 Cell Analyzer (Bio‐Rad) and the data were analyzed using FlowJo 10.5 software.

##### Preparation of PEI Conjugates and Nanovaccines

For PEI–Adpgk, 10 mg of PEI dissolved in 1 mL DMSO was mixed with SPDP crosslinker and stirred for 3 h, followed by the addition of CSS‐Adpgk. The amount of SPDP/CSS‐Adpgk was 1.3/1.1, 3.2/2.9, 6.4/5.8 µmol for PEI–Adpgk(2), PEI–Adpgk(13), PEI–Adpgk(30), respectively. After overnight reaction, PEI–Adpgk(2) remained dispersed while PEI–Adpgk(13) and PEI–Adpgk(30) formed off‐white particulates. To get rid of unreacted SPDP and CSS‐Adpgk, the crude mixture of PEI–Adpgk(2) was dialyzed 3 times against deionized (DI) water using Amicon ultra 10 kDa *M*_w_ cutoff centrifugal filters, while PEI–Adpgk(13) and PEI–Adpgk(30) were washed 3 times with DMSO by successive centrifugations. For PEG‐PEI‐antigen, PEI was first conjugated with Methoxy‐PEG‐NHS at varying stoichiometry, followed by antigen conjugation using SPDP crosslinker. Briefly, 5 mg of PEI dissolved in 1 mL DMSO was reacted overnight with 5, 10, 15, 20 mg of Methoxy‐PEG‐NHS for PEG(5)‐PEI, PEG(10)‐PEI, PEG(15)‐PEI, PEG(20)‐PEI, respectively. The conjugation was quantified by measuring primary amine contents of PEI using 2,4,6‐trinitrobenzene sulfonic acid according to the manufacturer's instruction. Then, SPDP/antigens were reacted as above with their amounts at 6.4/5.8 µmol. Antigen peptides employed for PEG‐PEI‐antigen conjugates include CSS‐Adpgk, CSSSIINFEKL, M27, and CSS‐M30. In some cases, 130 µg of Alexa Fluor® 488 NHS Ester (AF488‐NHS, Invitrogen) was added along with SPDP for fluorophore labeling of PEI. The crude mixtures were remained dispersed and purified by 3 rounds of dialysis using Amicon ultra 10 kDa *M*_w_ cutoff centrifugal filters. The final products were freeze‐dried and then re‐dispersed in DI water at 5 or 2 mg mL^−1^. For fluorophore labeling of CpG, 5′ phosphate group of CpG was first tethered with ethylenediamine via the 1‐ethyl‐3‐(3‐dimethylaminopropyl)carbodiimide coupling reaction in methyl imidazole buffer, followed by reaction with Alexa Fluor® 647 NHS Ester (AF647‐NHS, Invitrogen) as described before.^[^ ^35^ ^]^ For the construction of nanovaccine, 15 µg of CpG dispersed in 50 µl PBS was quickly added to 7.5, 15, 30, or 45 µg of PEI conjugates diluted in 50 µl PBS for weight ratio of PEI conjugate/CpG 0.5, 1, 2, 3, repectively. The solutions were vigorously mixed for 1 min at room temperature and stored at 4 °C before use.

##### In Vitro Cell Experiments

BMDCs were collected from C57BL/6 mice and maintained in the medium of RPMI 1640 supplemented 10% fetal bovine serum, 1% penicillin–streptomycin, 20 ng mL^−1^ granulocyte macrophage colony‐stimulating factor (Genscript), and 50 × 10^−6^ m *β*‐mercaptoethanol according to the literature.^[^ ^36^ ^]^ Immature BMDCs were plated at a density of 1 × 10^5^ cells/well in 96 well plates and incubated overnight at 37 °C under 5% CO_2_. For the cytotoxicity study, BMDCs were incubated with PEI–Adpgk conjugates or CSS‐Adpgk for 24 h, with the dose at 1, 5, 10, 20, 50, 100 µg mL^−1^. Then, Cell Counting Kit‐8 solution was added to each well of the plate according to manufacturer's instruction (Dojindo Laboratories, Japan). After 2 h, absorbance at 450 nm was measured using a microplate reader to calculate relative viability as the ratio of the absorbance to the nonsample treated cells. For the cellular uptake study, BMDCs were incubated with PEI–Adpgk/AF488 conjugates or their NPs with CpG‐AF647 at dose of 20 µg mL^−1^ PEI conjugates (10 µg mL^−1^ for free Adpgk) and/or 10 µg mL^−1^ CpG. At the indicated time points, cells were collected, washed with FACS buffer (1% BSA in PBS), and then subjected to flow cytometry for measuring fluorescence signals. To visualize cellular localization, BMDCs were grown onto 12 mm glass coverslips in 24 well plates at a density of 5 × 10^5^ cells/well and treated with samples as above for 24 h. Cells were further incubated with Hoechst 33342 (5 µg mL^−1^, Invitrogen) and Lysotracker Red DND‐99 (100 nM, Invitrogen) for 30 min for the staining of nuclei and endolysosomes, respectively. Then, cells were fixed with 4% formaldehyde in PBS and mounted on slide glass using ProLong™ Diamond Antifade Mountant (Invitrogen) for confocal microscopy (Nikon A1Rsi). For TLR‐9 signaling study, HEK‐blue TLR‐9 cells (Invivogen) were treated with PEI–Adpgk conjugates or their NPs at the dose of 2 µg mL^−1^ PEI conjugates and 1 µg mL^−1^ CpG in HEK‐Blue Detection medium. After 8 h, absorbance at 650 nm was measured using a microplate reader to analyze induction of TLR‐9 signaling in the cells, with the correction of the sample effect by subtracting the absorbance of samples without TLR‐9 cells. For the analysis of activation and antigen cross‐presentation, BMDCs were incubated with PEI‐SIINFEKL conjugates or their NPs for 24 h, with the dose at 2 µg mL^−1^ PEI conjugates or free SIINFEKL and 1 µg mL^−1^ CpG. Cells were collected, washed with FACS buffer, incubated with CD16/32 FcR blocking antibody (Invitrogen, No. 14016186) for 10 min, and then stained with antibody‐fluorophore conjugates including CD80‐FITC (BD Biosciences, No. 553768), CD86‐PE/Cy7 (BD Biosciences, No. 560582), CD40‐APC (Invitrogen, No. 17040182) and SIINFEKL/H‐2k^b^‐PE (Invitrogen, No. 12574382) for 30 min at room temperature. After wasing with FACS buffer, cell were analyzed using flow cytometry. To visualize antigen cross‐presentation, BMDCs were grown onto 12 mm glass coverslips in 24 well plates at a density of 5 × 10^5^ cells/well, treated with samples for 24 h, and further incubated with Hoechst 33342 for 30 min. Then, cells were incubated with CD16/32 FcR blocking antibody, permeabilized with Cytofix/Cytoperm Fixation/Permeabilization Solution (BD Biosciences), and antibody‐stained with SIINFEKL/H‐2k^b^‐biotin (Invitrogen, No. 13574381) and LAMP1‐AF488 (Invitrogen, No. 53107182). After further staining with streptavidin‐AF594 (Molecular Probes, No. S32356), cells were washed with PBS and mounted on slide glass using ProLong™ Diamond Antifade Mountant for confocal microscopy.

##### In Vivo Tumor Retention and Lymph Node Draining Studies

Animals were cared for following the federal, state, and local guidelines. The University of Michigan, Ann Arbor is an AAALAC international accredited institution, and all work conducted on animals was in accordance with and approved by the Institutional Animal Care and Use Committee (IACUC) with the protocol # PRO00008587. Female C57BL/6 mice (5–6 weeks) were purchased from Jackson Laboratory (USA). C57BL/6 mice were subcutaneously inoculated with 5  ×  10^5^ MC38 cells into the right flank and randomly sorted for treatment after 9 days when tumor size reached approximately 5 mm. The mice were administered intratumorally with 50 µl PBS solution of Adpgk vaccine formulations with CpG‐AF647 at the dose of 30 µg PEI–Adpgk conjugates (equivalent of 10 µg for free Adpgk) and 15 µg CpG. For the analysis of tumor retention, tumors were excised 24 h after sample administration and their fluorescence intensity was measured using IVIS optical imaging system (Caliper Life Sciences). For uptake in a cellular level, tumors were cut into small pieces, incubated with 1  mg mL^−1^ of collagenase type IV and 0.1 mg mL^−1^ of DNase I in RPMI for 30 min at 37 °C, and filtered through a 70‐µm strainer. The obtained single cell suspension was washed with FACS buffer, and their fluorescence signal was measured using flow cytometry. For the analysis of DCs and macrophages in lymph node, inguinal and axillary lymph nodes were collected 24 h after sample administration, ground with the rubber end of a syringe, and filtered through a 70‐µm strainer. The cell suspension was washed with FACS buffer, incubated with CD16/32 FcR blocking antibody, and stained with the following antibody‐fluorophore conjugates; CD80‐FITC (BD Biosciences, No. 553768), CD40‐PE (Invitrogen, No. 12040183), CD86‐PE/Cy7 (BD Biosciences, No. 560582), CD11c‐APC (BioLegend, No. 117309) for CD11c+ DCs, and CD11b‐PE (Invitrogen, No. 12011282), F4/80‐APC (BioLegend, No. 123116), CD86‐PE/Cy7 (BD Biosciences, No. 560582), CD206‐APC/Cy7 (BioLegend, No. 321120) for CD11b+F4/80+ macrophages. All flow cytometry was performed after suspending cells in DAPI solution for counting only DAPI‐negative live and intact cells.

##### In Vivo Cancer Therapy

For MC38 tumor study, C57BL/6 mice were subcutaneously inoculated with 5  ×  10^5^ MC38 cells into the right flank and randomly sorted for treatment after 9 days. The mice were intratumorally administered with 50 µl PBS solution of Adpgk vaccine formulations at the dose of 30 µg PEI–Adpgk conjugates (equivalent of 10 µg for free Adpgk) and 15 µg CpG. In some cases, samples were administered into tail‐base subcutaneous site for subcutaneous injection or tail‐vein for intravenous injection (100 µl in PBS). For analysis of neoantigen‐specific CD8^+^ T cells in systemic circulation, submandibular bleeding was performed at the indicated time points and PBMCs were collected after removing red blood cells using ACK lysis buffer. PBMCs were incubated with CD16/32 FcR blocking antibody and then stained with Adpgk peptide‐MHC tetramer tagged with PE (H‐2D^b^‐restricted ASMTNMELM, NIH Tetramer Core Facility) and anti‐CD8‐APC (BD Biosciences, No. 553035). For analysis of tumor infiltrating lymphocytes, tumor tissues were collected 7 days after sample administration, cut into small pieces, treated with 1 mg mL^−1^ of collagenase type IV and 0.1 mg mL^−1^ of DNase I in RPMI for 30 min at 37 °C, and filtered through a 70‐µm strainer. Then, the cell suspension was washed with FACS buffer, incubated with CD16/32 FcR blocking antibody, and stained with the following antibody‐fluorophore conjugates; Perforin‐FITC (Invitrogen, No. 11939280), Granzyme‐PE/Cy7 (Invitrogen, No. 25889882), CD45‐PerCP/Cy5.5 (Invitrogen 45045182), Adpgk peptide‐MHC tetramer‐PE, CD8‐APC for CD8^+^ T cells, Perforin‐FITC, Granzyme‐PE/Cy7, CD45‐PerCP/Cy5.5, NK1.1‐PE (Invitrogen, 12594182) for NK cells, and CD45‐PerCP/Cy5.5, Foxp3‐PE/Cy7 (Invitrogen, No. 25577382) CD4‐APC (Invitrogen, No. 17004282) for CD4^+^ T cells and CD4^+^Foxp3+ Tregs. Flow cytometry was performed after suspending cells in DAPI solution and gating out DAPI‐positive populations. For B16F10 tumor study, C57BL/6 mice were injected with 3  ×  10^5^ B16F10 cells subcutaneously into the right flank and 4  ×  10^5^ B16F10 cells intravenously into tail vein, for locally established tumors and lung metastasis, respectively. The subcutaneous tumors were subjected to intratumoral administration of samples in 50 µl PBS, with the dose at 15 µg PEI‐M27 conjugate (equivalent of 3.5 µg for free M27) and 7.5 µg CpG for M27 vaccine and 15 µg PEI‐M27 conjugate, 15 µg PEI‐M30 conjugate (equivalent of 5.5 µg for free M30), and 15 µg CpG for M27/M30 vaccine. Animals were randomly sorted on day 7 and received samples every 3 days for total 3 times, followed by euthanization on day 17 for the analysis of splenocyte ELISPOT and lung metastasis. For ELISPOT assay, spleens were ground with the rubber end of a syringe, filtered through a 70‐µm strainer, and treated with ACK lysis buffer for removing red blood cells. The obtained splenocytes were plated at 2 × 10^5^ cells/well in 96‐well PVDF plates pre‐coated with IFN‐*γ* antibody (BD Biosciences), and re‐stimulated overnight with 10 µg mL^−1^ of M27 or M30 peptide. Then, the wells were sequentially treated with biotinylated‐secondary antibody, streptavidin alkaline phosphatase, and AEC Substrate (BD Biosciences). The developed spots were counted using AID iSpot Reader (AID GmbH, Germany). For the analysis of lung metastasis, lungs were excised, fixed overnight in 4% formaldehyde, and then B16F10 lung tumor nodules were enumerated manually. The sizes of locally established tumors were measured twice a week using a digital caliper, and the tumor volume was estimated by ellipsoidal calculation as V  =  (width)^2^  ×  length  ×  1/2. The mice were euthanized when the tumors reached the maximum permitted size (1.5 cm in any dimension) or ulcerations occurred.

##### Statistical Analysis

For animal studies, the mice were randomized to match similar average volume of the locally established tumors. The data show mean  ±  s.d. (n = 5–8).  Data were approximately normally distributed and variance was similar between the groups. Statistical analysis was performed with Prism 8.1.0 software (GraphPad Software) by one‐way or two‐way ANOVA with Bonferroni multiple comparisons post‐test. Statistical significance for survival curve was calculated by the log‐rank (Mantel–Cox) test. All data were included for the statistical analysis with the significance indicated as *P  <  0.05, **P  <  0.01, ***P  <  0.001, and ****P  <  0.0001.

## Conflict of Interest

The authors declare no conflict of interest.

## Supporting information

Supporting Information
