# Supplementary material for: Modularly Programmable Nanoparticle Vaccine Based on Polyethyleneimine for Personalized Cancer Immunotherapy
Source: Adv Sci (Weinh). 2021 Jan 6;8(5):2002577. doi: 10.1002/advs.202002577 (PMC7927624; doi:10.1002/advs.202002577)
Supplement: Supplementary file 1 — Supporting Information [file ADVS-8-2002577-s001.pdf]

WILEY-VCH

© 2020 Wiley-VCH GmbH

ADVANCED SCIENCE
Open Access

Supporting Information

for *Adv. Sci.,* DOI: 10.1002/advs.202002577

Modularly programmable nanoparticle vaccine based on polyethyleneimine for personalized cancer immunotherapy

*Jutaek Nam, Sejin Son, Kyung Soo Park, and James J. Moon**

Supporting Information

Modularly programmable nanoparticle vaccine based on polyethyleneimine for personalized cancer immunotherapy

*Jutaek Nam, Sejin Son, Kyung Soo Park, and James J. Moon**

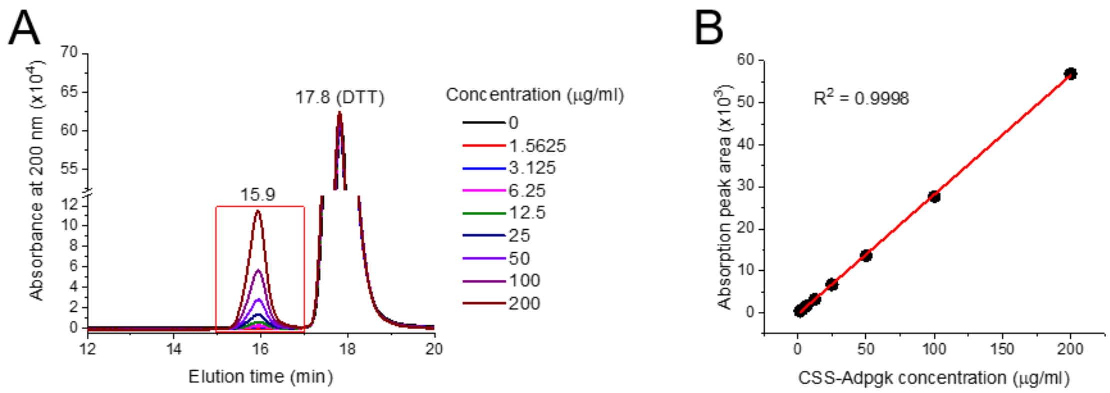

Figure S1. GPC spectra of CSS-Adpgk peptides obtained after 10 mM DTT treatment (A) and corresponding standard curve of concentration vs. absorption peak area (B).

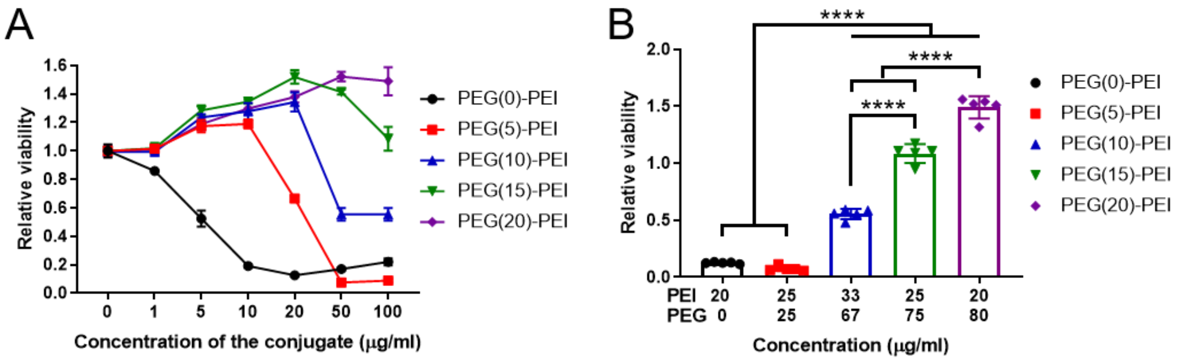

Figure S2. Relative viability of BMDCs after 24 h incubation with PEG-PEI conjugates in a concentration range of 1 – 100 μg/ml (A) and the viability compared for 20 μg/ml PEG(0)-PEI, 50 μg/ml PEG(5)-PEI, and 100 μg/ml PEG(10)-, PEG(15)-, PEG(20)-PEI with their PEI and PEG content separately marked on the x-axis (B).

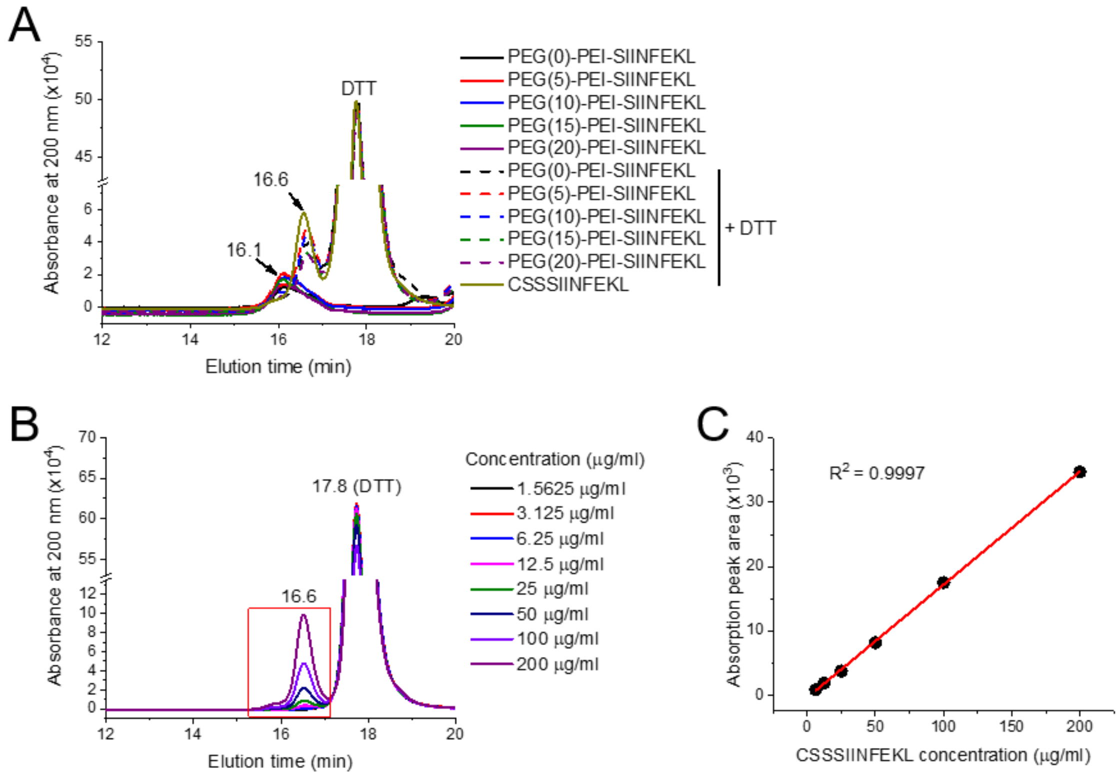

Figure S3. GPC spectra of PEG-PEI-SIINFEKL conjugates obtained before and after 10 mM DTT treatment (A), CSS-SIINFEKL peptides after 10 mM DTT treatment (B), and the standard curve of concentration vs. absorption peak area for CSS-SIINFEKL (C).

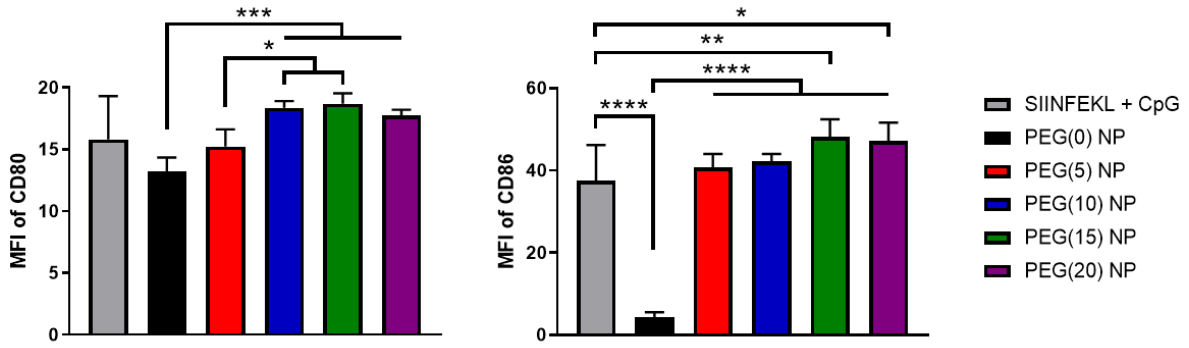

Figure S4. MFI of CD80 and CD86 co-stimulatory markers expressed on BMDCs after incubation with SIINFEKL + CpG or SIINFEKL nanovaccines.

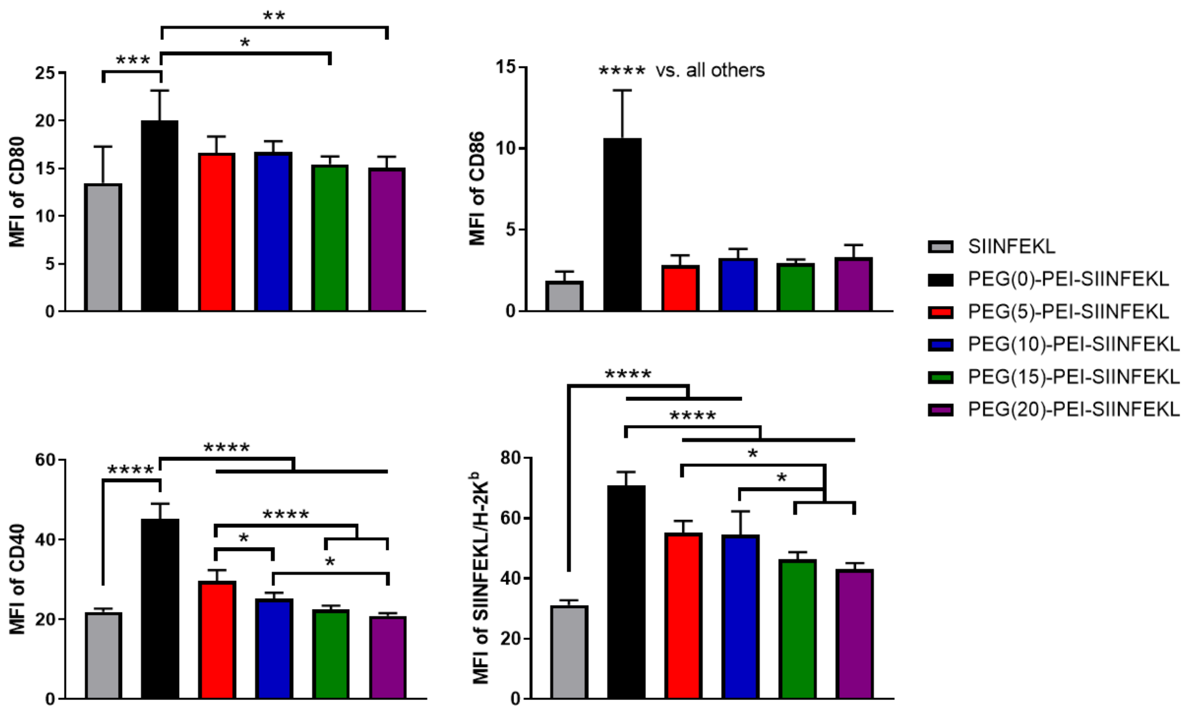

Figure S5. Upregulation of CD80, CD86, CD40, and SIINFEKL/H-2K^b by BMDCs after 24 h incubation with SIINFEKL or PEG-PEI-SIINFEKL conjugates.

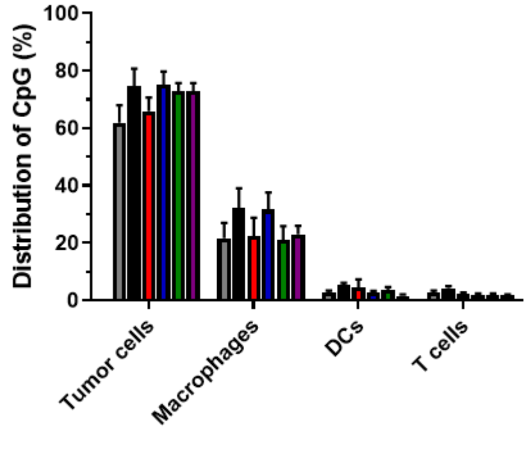

Figure S6. Cellular distribution of CpG in tumor tissues.

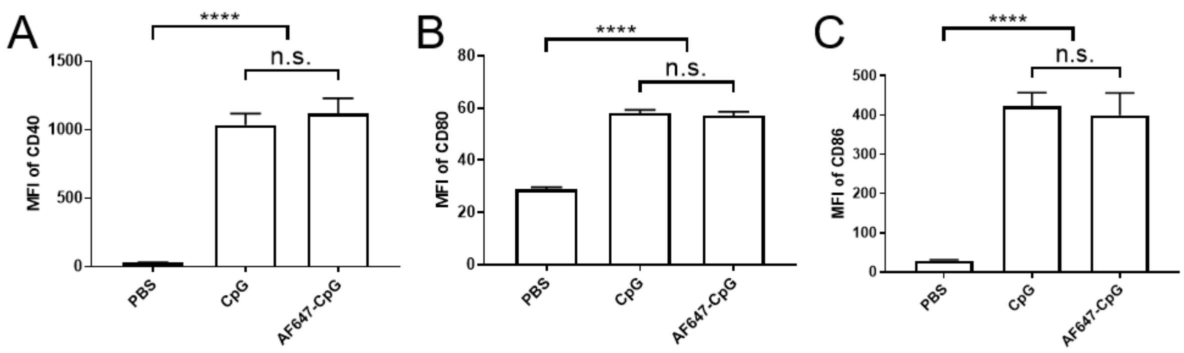

Figure S7. MFI of CD40 (A), CD80 (B), CD86 (C) co-stimulatory markers expressed on BMDCs measured after 24 h incubation with CpG or AF647-CpG conjugate at CpG concentration of 1 µg/ml.

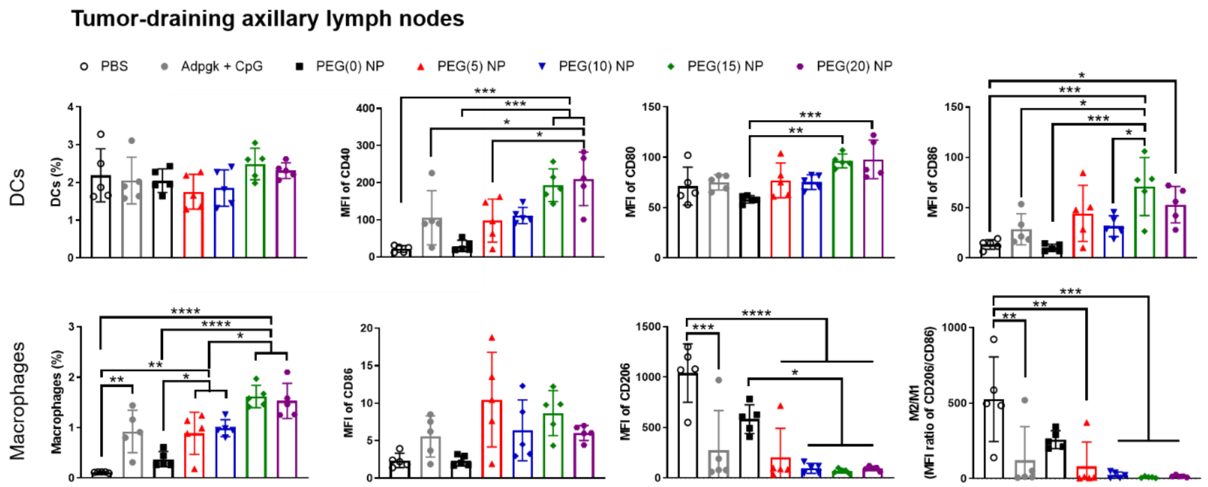

Figure S8. The percentage and activation of dendritic cells and macrophages in tumor-draining axillary lymph nodes.

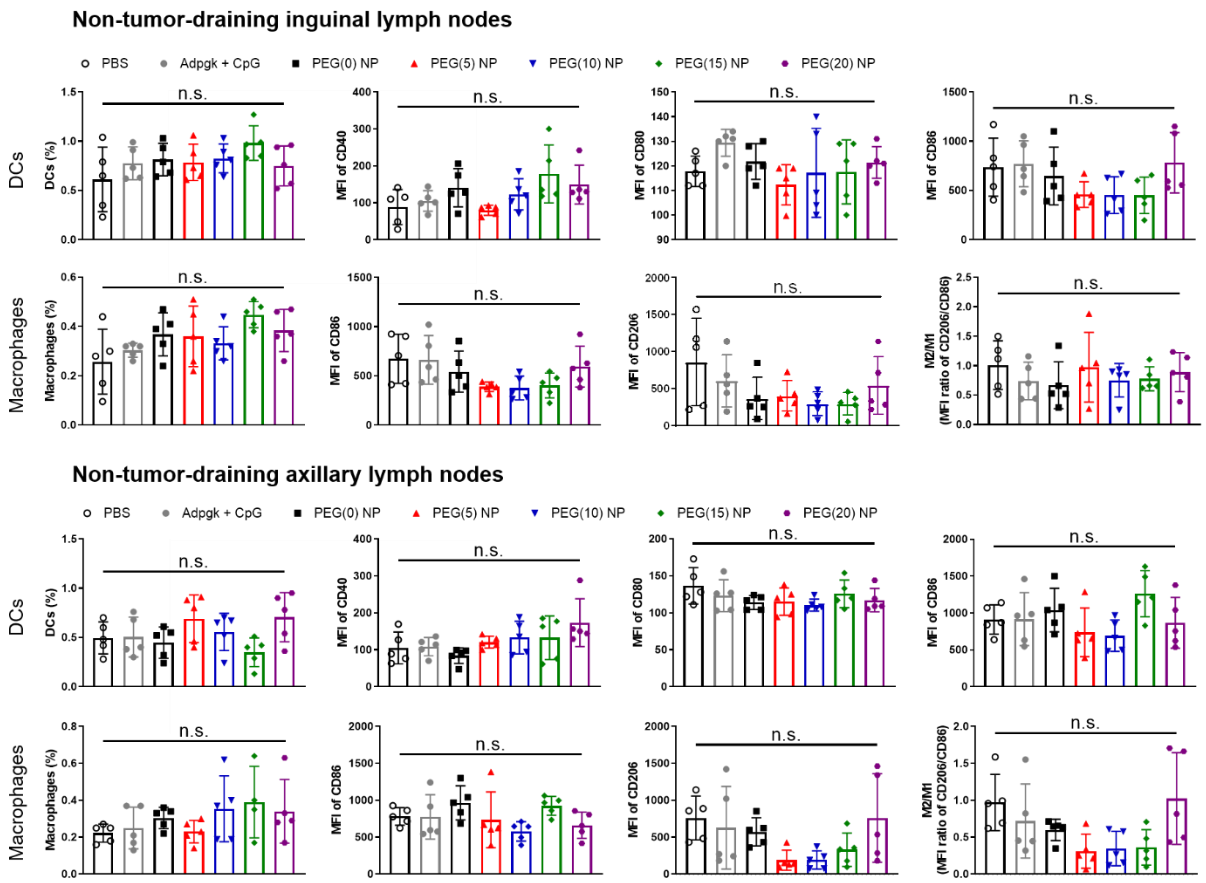

Figure S9. The percentage and activation of dendritic cells and macrophages in contralateral non-tumor-draining inguinal and axillary lymph nodes.

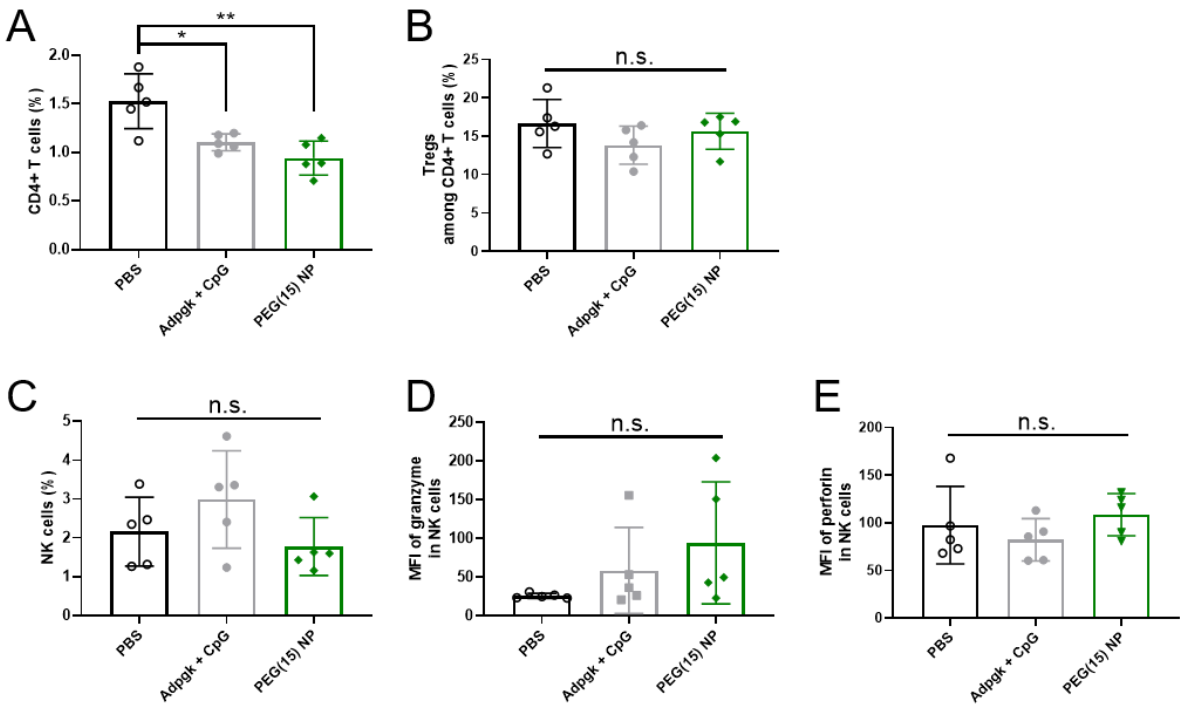

Figure S10. Tumor microenvironment analysis for the frequency of CD4+ T cells (A), regulatory T cells (B), NK cells (C), and MFI of granzyme (D) and perforin (E) in total NK cells.

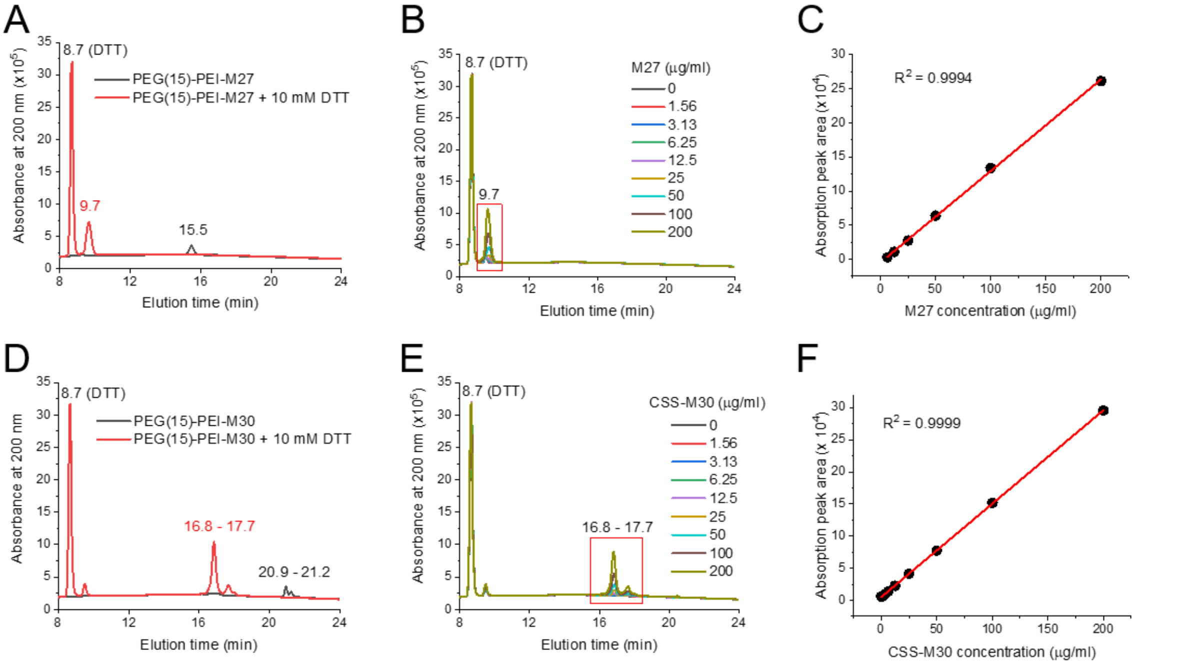

Figure S11. HPLC spectra of PEG(15)-PEI-M27 (A) and PEG(15)-PEI-M30 (D) obtained before and after 10 mM DTT treatment, M27 peptides (B) and CSS-M30 peptides (E) after 10 mM DTT treatment, and the standard curve of concentration vs. absorption peak area for M27 peptides (C) and CSS-M30 peptides (F).
